# Genomic and expression analysis of the flax (*Linum usitatissimum*) family of glycosyl hydrolase 35 genes

**DOI:** 10.1186/1471-2164-14-344

**Published:** 2013-05-23

**Authors:** Neil Hobson, Michael K Deyholos

**Affiliations:** 1 Department of Biological Sciences, University of Alberta, Edmonton, AB, T6G 2E9, Canada

**Keywords:** Flax, Industrial crop, β-galactosidase, Expression analysis, Phylogenetics

## Abstract

**Background:**

Several β-galactosidases of the Glycosyl Hydrolase 35 (GH35) family have been characterized, and many of these modify cell wall components, including pectins, xyloglucans, and arabinogalactan proteins. The phloem fibres of flax (*Linum usitatissimum*) have gelatinous-type cell walls that are rich in crystalline cellulose and depend on β-galactosidase activity for their normal development. In this study, we investigate the transcript expression patterns and inferred evolutionary relationships of the complete set of flax GH35 genes, to better understand the functions of these genes in flax and other species.

**Results:**

Using the recently published flax genome assembly, we identified 43 β-galactosidase-like (BGAL) genes, based on the presence of a GH35 domain. Phylogenetic analyses of their protein sequences clustered them into eight sub-families. Sub-family B, whose members in other species were known to be expressed in developing flowers and pollen, was greatly under represented in flax (p-value < 0.01). Sub-family A5, whose sole member from arabidopsis has been described as its primary xyloglucan BGAL, was greatly expanded in flax (p-value < 0.01). A number of flax BGALs were also observed to contain non-consensus GH35 active sites. Expression patterns of the flax BGALs were investigated using qRT-PCR and publicly available microarray data. All predicted flax BGALs showed evidence of expression in at least one tissue.

**Conclusion:**

Flax has a large number of BGAL genes, which display a distinct distribution among the BGAL sub-families, in comparison to other closely related species with available whole genome assemblies. Almost every flax BGAL was expressed in fibres, the majority of which expressed predominately in fibres as compared to other tissues, suggesting an important role for the expansion of this gene family in the development of this species as a fibre crop. Variations displayed in the canonical GH35 active site suggest a variety of roles unique to flax, which will require further characterization.

## Background

In 1894, an enzyme preparation was found to catalyze lactose hydrolysis [1], initiating the study of proteins we have come to know as β-D-galactoside galactohydrolases (β-galactosidases). In the proceeding decades, a β-galactosidase protein was purified from *Escherichia coli* for kinetic studies [2], and *LacZ*, a bacterial gene coding for a β-galactosidase, was characterized during a seminal examination of the *lac* operon and transcriptional regulation [3].

β-Galactosidases (EC 3.2.1.23) hydrolyze the terminal non-reducing β-D-galactose residues in β-D-galactosides, such as lactose, proteoglycans, glycolipids, oligosaccharides, and polysaccharides [4]. Other classes of enzymes are known to hydrolyze bonds involving galactose residues (EC 3.2.1.85; EC 3.2.1.89; EC 3.2.1.102; EC 3.2.1.103; EC 3.2.1.145; EC 3.2.1.164; EC 3.2.1.18), however, the nature of the substrate and/or reaction mechanism of these enzymes is sufficiently different from EC 3.2.1.23 BGALs as to render these enzyme classes distinct [4].

Distributed across kingdoms, β-galactosidases are represented in bacteria, fungi, plants and animals. Based on sequence and structural similarity, EC 3.2.1.23 β-galactosidases can be placed in five of the current 131 glycosyl hydrolase (GH) families: GH1, GH2, GH3, GH35, and GH42 [5]. Plant β-galactosidases have been found only in GH35; β-galactosidases from the other four families have been observed solely in bacteria and archaea. Henceforth, we will use the term BGAL to refer to any GH35 β-galactosidase-like gene.

In plants, BGALs have been found to play a role in: the degradation of cell wall polysaccharides; promoting fruit softening [6,7]; organization of cellulose microfibrils in fibre cells [8,9]; promoting cell elongation [10]; and facilitating the secretion of seed mucilage [11].

The BGALs of flax (*Linum usitatissimum*) have not been well studied. To date, only a single flax β-galactosidase (LuBGAL1) has been characterized, which has an important role in the development of cell walls of phloem fibres [8]. The recent publication of a draft flax genome sequence [12] now allows researchers to study industrially relevant gene families in their entirety, such as the previously reported analysis of the UDP glycosyltransferase 1 family [13]. We describe here a detailed analysis of the primary structure, evolutionary history, and transcript expression patterns of 43 putative β-galactosidases in flax.

## Methods

### Gene discovery

The 43,384 predicted proteins of the flax genome [12], available at Phytozome (version 8.0) [14], were first queried via BLASTP for sequences similar to the 17 known arabidopsis BGALs (AtBGALs 1-17; TAIR v.10) [15]. The default settings of BLAST package 2.2.25+ were used. Sequence matches were filtered for e-values ≤ 1^-10^, and then assessed via Hidden Markov Model (HMM) with HMMER3 [16], using the Pfam-A family database (version 25.0) [17], for genes encoding a glycosyl hydrolase 35 domain (GH35). Comparisons of gene family size were performed with a one-tailed Z-test of proportions.

### Phylogenetics

Predicted protein sequences from *Arabidopsis thaliana*, *Oryza sativa*, *Physcomitrella patens*, *Populus trichocarpa*, *Ricinus communis*, and *Zea mays* were obtained from Phytozome (version 8.0) [14,18-22]. Sequences were assessed via Hidden Markov Model (HMM) with HMMER3 [16], using the Pfam-A family database (version 25.0) [17], for genes putatively encoding a GH35 domain. Retrieved sequences were labelled as BGALs (Additional file 1: Table S1), using published BGAL names (e.g. AtBGAL1) wherever possible [23,24]. Amino acid sequences were aligned using the default parameters of Muscle 3.7 [25], with a human beta-galactosidase (GLB1), obtained from NCBI genbank (NP_000395), as an outgroup. ProtTest 3.2, with default parameters, was used to determine the best-fit model of amino acid substitution for a maximum likelihood analysis of the sequence alignment [26]. Using the WAG model of amino acid substitution [27], while employing gamma-distributed rate variations, we performed a maximum likelihood analysis with GARLI [28-30]. The consensus tree of 1000 bootstraps was obtained using CONSENSE (Phylip 3.66) at the CIPRES Science Gateway [31].

### EST identification

Genomic sequence of putative flax BGALs, including 1 kb upstream and downstream of their respective start and stop codons, were used as queries in a BLASTN search against the *Linum usitatissimum* NCBI-nr and NCBI-EST datasets (accessed August, 2012), as well as transcript assembly POZS [32], comprising a *de novo* assembly of Illumina sequenced transcripts from three flax stem fragments. All sequence matches were downloaded and aligned to the predicted LuBGAL CDSs using the RNA-SEQ analysis tool of CLC Genomics Workbench 5.5. Only sequences aligning to CDSs with 95% identity, along 90% of their length, were recorded.

### Microarray analyses

Flax microarray datasets GSE21868 [33] and GSE29345 [34] were obtained from NCBI GEO. Experiment GSE21868 examined expression in a range of tissues and organs: roots (R); leaves (L); outer stem tissues at either the vegetative stage (SOV) or green capsule stage (SOGC); inner stem tissues at either vegetative stage (SIV) or green capsule stage (SIGC); and seeds 10-15 days after flowering (DAF; E1), 20-30 DAF (E2), and 40-50 DAF (E3) [33]. Experiment GSE29345 focused on the development of stem tissues by comparing: internal (i.e. xylem enriched) stem tissues of either the whole stem (WSI), upper stem (USI), middle stem (MSI), or lower stem (LSI); and external (i.e. phloem and cortex enriched) stem tissues of the whole stem (WSE), upper stem (USE), middle stem (MSE), and lower stem (LSE) [34]. The flax unigenes used in microarray construction [35] were aligned to the predicted *LuBGAL* CDSs, using the RNA-Seq function of the CLC Genomics Workbench 5.5, and were classified as matches if at least 90% of their sequence length aligned to a genomic fragment, with at least 95% sequence identity between the transcript and CDS. Microarray data corresponding to the flax BGALs were then extracted. Robust Multichip Average (RMA)-normalized signal intensities (log_2_) were averaged between biological and technical replicates. Heat maps of expression levels were then created with MeV v4.8 [36].

A Combimatrix microarray dataset examining five stages of flax stem development was produced in our laboratory (manuscript in preparation). The array profiled 1 cm stem fragments from the shoot apex (T1), sections of the snap-point corresponding to various stages of fibre development (T2-4), and lower stem with phloem fibres exhibiting a greater degree of secondary cell wall deposition (T5). Probes, 33-40 nt in length, corresponding to predicted *LuBGALs* from an earlier draft of the flax genome (unpublished) were aligned to the current *LuBGAL* CDS predictions (version 1.0) [12] using the RNA-Seq function of CLC Genomic Workbench 5.5. Only probes with 100% identity to existing *LuBGAL* CDSs were analyzed. Gene signal intensities were normalized as fractions of mean array signal intensity. The log_2_ normalized *LuBGAL* intensities, averaged between four biological replicates, were then used to create heat maps of expression levels with MeV v4.8 [36].

### Expression analysis of LuBGALs

Tissue samples from *Linum usitatissimum* (CDC Bethune) were frozen in liquid nitrogen, and stored at -80°C prior to use. Frozen samples were ground in liquid nitrogen, whereupon we followed the CTAB/Acid Phenol/Silica Membrane Method [37] to extract the RNA. DNA was removed using on-column RNase-Free DNase (Qiagen), and/or with the TURBO DNA-Free kit (Invitrogen). cDNA was prepared with RevertAid H Minus Reverse Transcriptase (Fermentas) and oligo(dT)_18_ primer. qPCR primer pairs and hydrolysis probes (Additional file 2: Table S2) were designed with the Universal Probe Library Assay Design Center [38]. A 14 cycle pre-amplification of the target sequences was performed with a TaqMan PreAmp Master Mix (ABI) and 5 ng of cDNA, which was subsequently diluted 1:5. Assay master mixes of 3.2 μl 2X Assay Loading Reagent (Fluidigm PN 85000736), 2 μl primer mix (13.3 μM primer and 3.3 μM hydrolysis probe) and 1.3 μl water was prepared, of which 5 μl was loaded into the assay wells of a primed Fluidigm 96*96 well chip. Sample master mixes of 3.63 μl Taqman Universal PCR Master Mix - no AmpErase UNG (PN 4324018), 0.36 μl 20X GE Sample Loading Reagent (Fluidigm PN 85000735), and 2.5 ul diluted pre-amped cDNA were prepared, of which 5 μl was loaded into the sample wells of the primed Fluidigm 96*96 well plate. The Fluidigm chip was run through the following thermal cycles: 95°C – 10 min, 40X cycles of 95°C – 15 sec and 60°C – 1 min. ΔC_T_ values were calculated based on the geometric mean of reference genes *ETIF1* (eukaryotic translation initiation factor 1), *GAPDH* (glyceraldehyde 3-phosphate dehydrogenase), and *ETIF5A* (eukaryotic translation initiation factor 5A) [39,40]. We compared expression in 12 different tissues: roots (R); leaves (L); senescing leaves (SL); stem apex (SA); cortical peels from vegetative stage stems (ECP) or green capsule stage stems (LCP); phloem fibres from vegetative stage stems (EF) or green capsule stage stems (LF); xylem from vegetative stage stems (X); budding flowers (FB); open flowers (F); and seed bolls from the green capsule stage (B). A heat map of relative expression values (log_2)_, averaging technical (two for F, FB, L, and SL; three for all other samples) and biological (three, each of which is a pooled sample from multiple plants) replicates, was then prepared with MeV v4.8 [36].

## Results

### Gene discovery and *in silico* analyses

**Figure 1:**
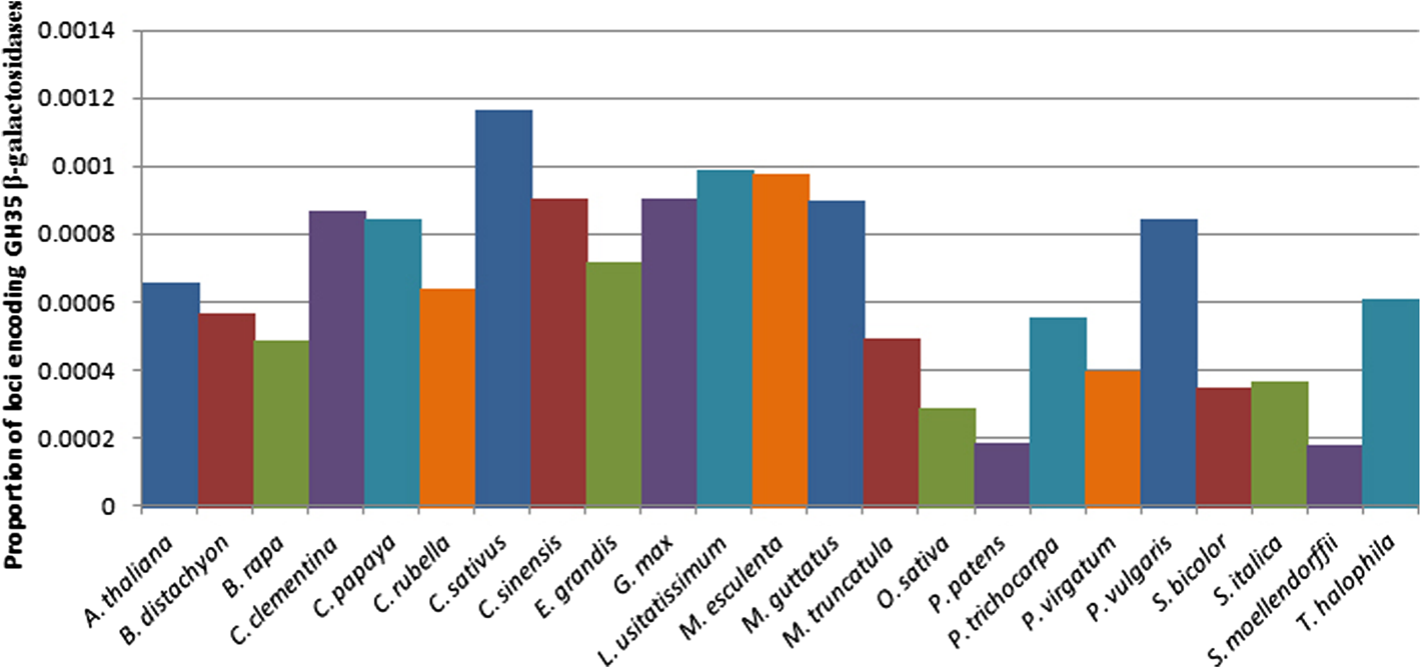
**Relative quantity of BGAL genes in the genomes of various plant species.** Predicted proteomes for *Arabidopsis thaliana*, *Brachypodium distachyon*, *Brassica rapa*, *Citrus clementina*, *Carica papaya*, *Capsella rubella*, *Cucumis sativus*, *Citrus sinensis*, *Eucalyptus grandis*, *Glycine max*, *Linum usitatissimum*, *Manihot esculenta*, *Mimulus guttatus*, *Medicago truncatula*, *Oriza sativa*, *Physcomitrella patens*, *Populus trichocarpa*, *Panicum virgatum*, *Phaseolus vulgaris*, *Sorghum bicolor*, *Setaria italica*, *Selaginella moellendorffii*, and *Thellungiella halophila* were obtained from Phytozome (version 8.0) [14]. Sequences were assessed via Hidden Markov Model (HMM) with HMMER3 [15], using the Pfam-A family database (version 25.0) [17], for genes putatively encoding a glycosyl hydrolase 35 domain. The number of putative BGAL genes was compared to the total number of protein coding loci published for each species at Phytozome (version 8.0) [14].

A combination of BLASTP searches and PFAM analyses resulted in the identification of 43 putative flax β-galactosidases (BGALs), on 34 separate scaffolds of the *de novo* flax genome assembly [12] (Table 1). Using the same approach for gene discovery, we compared the size of the flax BGAL families in 23 representative plant genomes obtained through Phytozome (version 8.0) [14]. We found that, relative to the number of protein coding loci in the genomes, flax had the second largest BGAL family, comprising 0.0989% of the total gene coding loci (Figure 1), significantly larger than the average BGAL family size (p-value < 0.01). In comparison, amongst the 23 species examined, the BGAL gene family represented an average of 0.0596% of the protein coding loci, or roughly 22 BGAL family members per species. The best-characterized examples include the BGAL families of *Arabidopsis thaliana* and *Oryza sativa*, for which 17 and 15 BGALs have been respectively described [23,24]. Even other members of the Malpighiales, such as *Populus trichocarpa* and *Ricinus communis,* contained half the number of BGALs as flax, at 23 and 21 members respectively (Additional file 1: Table S1).

**Table 1:** Summary of glycosyl hydrolase 35 encoding gene homologues

**Gene name**	**Genomic contig**	**Gene ID**	**mRNA**^**a**^	**ESTs**^**a**^	**Scaffold gap (bp)**
LuBGAL1	scaffold1486	Lus10008974.g	1	3	N
LuBGAL2	scaffold540	Lus10028848.g		4	N
LuBGAL3	scaffold328	Lus10006009.g		16	N
LuBGAL4	scaffold156	Lus10040557.g		5	N
LuBGAL5	scaffold504	Lus10000701.g		0	N
LuBGAL6	scaffold630	Lus10015625.g		8	N
LuBGAL7	scaffold196	Lus10037644.g		6	N
LuBGAL8	scaffold1252	Lus10000803.g		0	N
LuBGAL9	scaffold16	Lus10024292.g		0	N
LuBGAL10	scaffold204	Lus10006733.g		1	N
LuBGAL11	scaffold1376	Lus10011237.g		0	N
LuBGAL12	scaffold275	Lus10014278.g		4	Y (494)
LuBGAL13	scaffold319	Lus10025980.g		4	N
LuBGAL14	scaffold3	Lus10020968.g		0	N
LuBGAL15	scaffold413	Lus10028348.g		4	N
LuBGAL16	scaffold272	Lus10041798.g		7	N
LuBGAL17	C8385757	Lus10000271.g		0	N
LuBGAL18	scaffold76	Lus10036109.g		0	N
LuBGAL19	scaffold915	Lus10016655.g		1	N
LuBGAL20	scaffold1120	Lus10003343.g		0	N
LuBGAL21	scaffold59	Lus10022645.g		3	N
LuBGAL22	scaffold305	Lus10025108.g		3	Y (8602)
LuBGAL23	scaffold305	Lus10025110.g		0	N
LuBGAL24	scaffold177	Lus10023977.g		6	N
LuBGAL25	scaffold177	Lus10023974.g		0	N
LuBGAL26	scaffold1982	Lus10005070.g		0	N
LuBGAL27	scaffold1143	Lus10027843.g		0	N
LuBGAL28	scaffold1247	Lus10014126.g		0	N
LuBGAL29	scaffold1982	Lus10005071.g		0	N
LuBGAL30	scaffold1143	Lus10027844.g		0	N
LuBGAL31	scaffold1247	Lus10014125.g		1	N
LuBGAL32	scaffold1491	Lus10019784.g		1	N
LuBGAL33	scaffold388	Lus10008259.g		0	Y (101 + 104 + 975)
LuBGAL34	scaffold711	Lus10020875.g		7	N
LuBGAL35	scaffold711	Lus10020877.g		1	N
LuBGAL36	scaffold701	Lus10033500.g		0	N
LuBGAL37	scaffold701	Lus10033502.g		0	N
LuBGAL38	scaffold112	Lus10018138.g		0	Y (16)
LuBGAL39	scaffold346	Lus10028538.g		0	N
LuBGAL40	scaffold488	Lus10033427.g		0	N
LuBGAL41	scaffold630	Lus10015616.g		6	N
LuBGAL42	scaffold196	Lus10037634.g		1	N
LuBGAL43	scaffold25	Lus10043422.g		0	N

^a^The number of mRNA and ESTs identified from the NCBI Genbank database and transcriptome assembly POZS [32].

To determine which of the predicted *LuBGAL* genes were expressed, we used BLASTN to align the *LuBGAL* CDS sequences with the NCBI-nr and NCBI-EST databases (accessed August 2012), and with *de novo* transcriptome assemblies of developing flax stems [32]. At the time of writing, the NCBI-EST database contained 286,852 sequences from *Linum usitatissimum*, 74.8% of which were obtained from flax seeds at various stages of development [41]. Ninety-three transcript sequences were identified, which aligned unambiguously to 21 of the *LuBGAL* CDSs (Table 1), indicating that EST data provided evidence for expression of approximately half the predicted LuBGAL family members. However, because only a limited number of tissues and conditions were represented by the EST sequences queried, it is likely that additional *LuBGALs* may also be expressed.

As described above, the predicted LuBGALs were defined by the presence of a GH35 domain, which was identified by alignment to PFAM HMM profiles. With one exception, in all of these proteins the GH35 domain was located near the N-terminus, beginning within the first 30-70 amino acids (Table 2). The one exception, LuBGAL24, contained a GH35 domain that started at position 568 of the peptide sequence, and was further distinguished by the presence of three N-terminal copper oxidase domains preceding the GH35 domain. The predicted LuBGALs were also searched for the presence of a GH35 active site [42], which contains the consensus sequence G-G-P-[LIVM](2)-x(2)-Q-x-E-N-E-[FY]. Two of the 43 predicted LuBGALs (LuBGAL35 and LuBGAL43) lacked the consensus active site entirely (Additional file 3: Figure S1). Another nine LuBGALs contained major deviations from the consensus active site; these either lacked the catalytic glutamate residues, as in LuBGAL26, or contained a series of insertions and substitutions in the active sites, as in LuBGALs 14, 20, 21, 22, 23, 24, 25, and 36. We note, however, that these deviations were not supported by ESTs. In addition to the GH35 domain, plant BGALs have occasionally been found to contain a putative galactose-binding lectin domain at the C-terminal end of the peptide sequence [23,24,43,44]. This cysteine rich domain has been proposed to increase the catalytic efficiency of BGAL proteins [23], and was found in only 22 of the 43 LuBGALs (Table 1), distributed roughly evenly amongst the different BGAL sub-families.

**Table 2:** Summary of predicted glycosyl hydrolase 35 protein homologues

**BGAL sub-family**	**LuBGAL**	**AA**	**MW**^**a **^**(kDA)**	**pI**^**a**^	**Signal peptide**^**b **^**(Cleavage Site)**	**Pfam domain**^**c**^			**Possible destinations (WolfPSORT)**^**d**^	**Possible destination (Plant-mPLOC)**^**e**^
						**GH35**	**Lectin**	**Copper oxidase**		
D	41	761	84.697	9.07	No	Y	N	N	cl, v, n, cy, m, pm	pm, cy
	42	701	78.278	8.06	No	Y	N	N	cl, n, er, cy	cw, cy
C1	32	816	91.547	9.03	No	Y	Y	N	cy, px, m, , n	cw
	31	756	84.239	9.07	No	Y	Y	N	cy, n, px, v	cw
	29	843	94.393	8.38	Yes (34-35)	Y	Y	N	cl, ex, v, n	cw
	30	833	93.226	7.42	Yes (24-25)	Y	Y	N	cl, ex, v, n	cw
	28	828	93.86	8.92	Yes (24-25)	Y	Y	N	v, cl, er, g, m, p	cw
	27	788	89.565	9.69	Yes (22-23)	Y	N	N	v, ex, er, g, cl	cw
	26	752	85.192	9.7	Yes (25-26)	Y	N	N	v, g, cl, ex, er	cw
C2	40	821	92.792	8.68	Yes (19-20)	Y	Y	N	er, pm, n, m, ex	cw
	38	810	91.236	9.06	Yes (24-25)	Y	N	N	er, v, g, cl, n, cy, pm	cw
	39	871	98.135	8.94	Yes (23-24)	Y	Y	N	v, er, g, cl, n, cy, pm	cw
	33	829	91.265	5.96	Yes (30-31)	Y	Y	N	n, er, pm, cl, cy	cw
	37	718	80.437	5.58	Yes (22-23)	Y	N	N	v, ex, er, g, cl	cw
	34	961	108.198	5.48	Yes (23-24)	Y	N	N	v, g, er	cw
	35	647	71.944	8.88	No	Y	N	N	n, cl, cy	cw
	36	706	79.027	8.79	No	Y	N	N	v, er, g, cl, n	cw
A1	9	727	81.545	8.69	Yes (26-27)	Y	N	N	cl, ex, n, v, er, g	cw
	8	683	76.432	8.72	Yes (25-26)	Y	N	N	cl, ex, er, pm, m, cy, v	cw
	13	849	94.313	6.62	Yes (29-30)	Y	Y	N	v, cy, pm, cl, n, ex	cw
	14	229	25.653	8.58	No	Y	N	N	cl, n, cy	pm, cl
	12	650	72.077	7.12	Yes (28-29)	Y	Y	N	v, er, ex, g, cl, cy	cw, pm
	16	849	94.704	7.37	Yes (30-31)	Y	Y	N	er, pm, cy, cl, n, m, p	cw
	15	802	89.416	6.65	Yes (30-31)	Y	Y	N	er, pm, n, cl, cy, m, px	cw
	5	844	93.587	6.79	Yes (29-30)	Y	Y	N	cl, ex	cw
	6	869	95.928	9.2	No	Y	Y	N	cl, v, g, n, pm	cw
	7	851	94.066	9.13	Yes (24-25)	Y	Y	N	cl, ex	cw
	4	717	80.14	9.16	Yes (23-24)	Y	N	N	cl, n	cw
	3	723	80.594	8.95	Yes (23-24)	Y	N	N	cl, ex	cw
	1	731	80.978	6.74	Yes (29-30)	Y	N	N	cl, ex	cw
	2	740	81.923	6.59	Yes (29-30)	Y	N	N	cl, ex	cw
A4	11	897	100.599	6.38	Yes (24-25)	Y	N	N	pm, g	cw
	10	854	94.48	5.31	Yes (24-25)	Y	Y	N	v, pm, er, g, cl	cw
	18	297	32.849	7.62	No	Y	N	N	m, cy, n, cl, pm, v, er	cl
	17	836	91.017	8.14	No	Y	Y	N	cy, v, n, m, pm, cl	cw
B	43	107	11.805	7.57	Yes (31-32)	Y	N	N	ex, v, cl, cy, m, er	pm
A5	22	1460	162.474	5.41	Yes (19-20)	Y	Y	N	ex, v, cl, n, pm	cw
	24	1330	147.844	8.24	Yes (23-24)	Y	Y	Y (3)	v, cl, n, pm, m, ex	pm, cw
	21	871	96.999	8.57	Yes (26-27)	Y	Y	N	er, n, pm, g, cy	cw
	20	874	97.552	8.75	Yes (26-27)	Y	Y	N	ex, v, er, g, cl, n, cy	cw
	23	718	80.588	5.3	Yes (19-20)	Y	N	N	ex, cl, v, cy	cw
	25	261	29.969	8.26	No	Y	N	N	n, cy, cl	cl
A2	19	880	98.216	6.52	Yes (27-28)	Y	Y	N	cl, v, g, pm	cw

^a^Predictions made with CLC Genomics Workbench 5.5.

^b^SignalP 4.0 prediction [45].

^c^Pfam domains and locations were identified with CLC Genomics Workbench 5.5.

^d^WolfPSORT prediction [46], in order of decreasing likelihood.

^e^Plant-mPLOC prediction [47].

Protein Destinations: cl (chloroplast), cy (cytosol), cs (cytoskeleton) cw (cell wall), er (endoplasmic reticulum), ex (extracellular), g (golgi apparatus), l (lysosome), m (mitochondria), n (nuclear), px (peroxisome), pm (plasma membrane), v (vacuolar membrane).

Unlike the described BGALs of rice [24] and arabidopsis [23], which are ~700-900 aa in length, the length of predicted flax BGALs was more variable in size (Table 2). Four putative flax BGALs (LuBGALs 14, 18, 25, and 43) were under 300 aa in length, while another two, LuBGALs 22 and 24, were greater than 1300 aa, with LuBGAL24 containing three copper oxidase domains at the N-terminus. Of these six atypically sized BGALs, only *LuBGAL22* and *LuBGAL24* are represented among ESTs or transcript assemblies (Table 1). In addition to the arabidopsis and rice BGAL genes previously described [23,24], we also identified an additional putative BGAL in each of these species, which we designated AtBGAL18 and OsBGAL16, respectively. AtBGAL18 was previously identified [23], but was not named. Both of these predicted proteins were less than 500 aa in length, and both lacked a consensus GH35 active site.

To determine the predicted subcellular localization patterns of the predicted LuBGALs, we analyzed the protein sequences for possible signal peptides, using SignalP 4.0 [45] (Table 2). We found that 32 of the 43 LuBGAL sequences contained a predicted signal peptide, generally located within the first 19-35 amino acids. The other 11 LuBGAL sequences, ranging in size from 229 to 869 aa, did not contain a signal peptide. We further employed WolfPSORT and Plant-mPLOC [46,47], and obtained a range of predicted subcellular destinations. In the case of Plant-mPLOC, proteins were predominantly predicted to localize to the cell wall, in some cases despite the lack of N-terminal signal peptide. Only eight LuBGALs were given alternative localization predictions, ranging from the cell membrane (LuBGALs 14, 24, 41, and 43), to the cytoplasm (LuBGALs 41, and 42) and chloroplast (LuBGALs 12, 14, 18, and 26). WolfPSORT was more variable in its predictions, with upwards of seven different predictions per putative LuBGAL. Predictions for the transport to the chloroplast and vacuoles were the most common, followed by the endoplasmic reticulum, extracellular space, and the cytoplasm. Surprisingly, a few LuBGALs were even predicted to most likely be localized to the nucleus (LuBGALs 25, 33, and 35). Experimental characterization will be required to validate these predictions.

### Phylogenetic analyses

**Figure 2:**
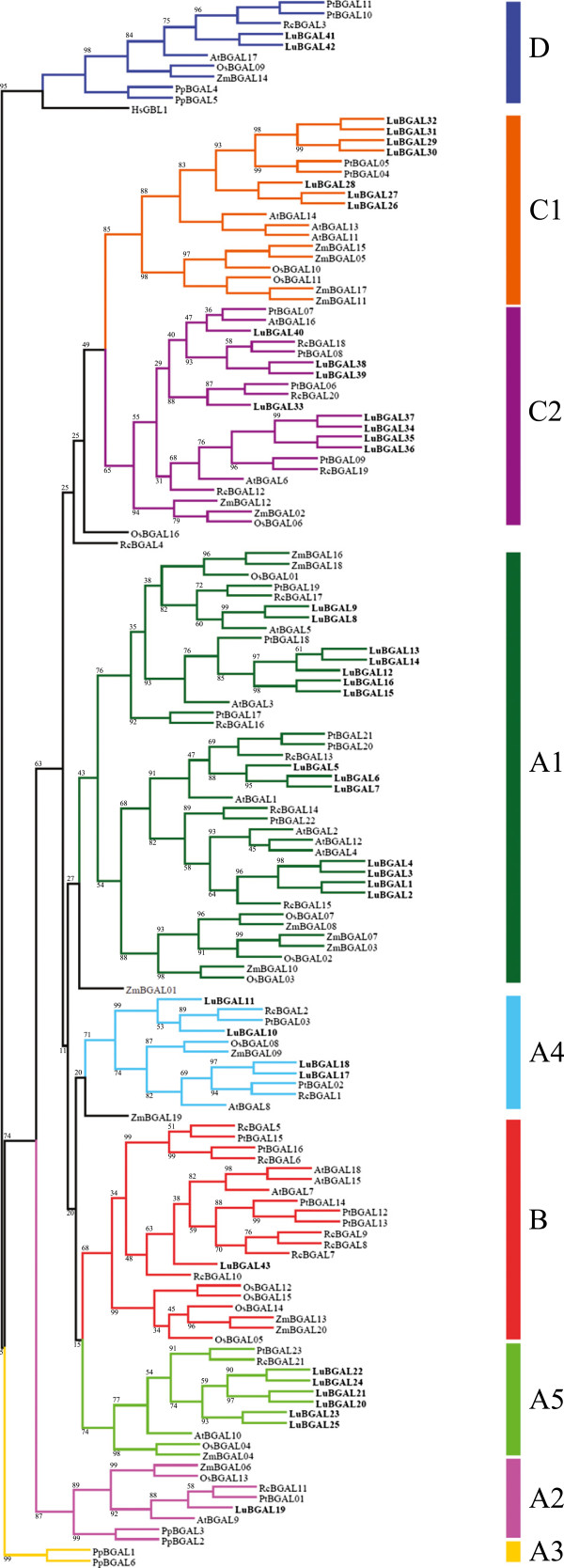
**Phylogenetic relationship among the glycosyl hydrolase 35 proteins of flax other species.** Deduced amino acid sequences were aligned with MUSCLE [25]. The tree was created with GARLI [28], using the maximum likelihood method, following the WAG model of amino acid substitutions [27]. A consensus tree of 1000 bootstrap replicates was produced for which percent reproducibility under 100 is shown. The flax sequences are named LuBGAL, and numbered according to Tables 1 and 2. *Arabidopsis thaliana* sequences are indicated as AtBGAL, and numbered according to existing designations [23]. *Oryza sativa* sequences are indicated as OsBGAL, and numbered according to existing designations [24]. *Physcomitrella patens* sequences are indicated as PpBGAL, *Populus trichocarpa* sequences are indicated as PtBGAL, and *Ricinus communis* sequences are indicated as RcBGAL. Genomic loci corresponding to these sequences are presented in Table 1. A human beta-galactosidase (GLB1; NP_000395) was used to establish the outgroup.

To classify LuBGALs based on sequence similarity, we performed a phylogenetic analysis using deduced amino acid sequences of the predicted BGAL coding genes from the genome assemblies of *L. usitatissimum*, *P. trichocarpa*, *R. communis*, *Physcomitrella patens*, *O. sativa*, *Zea mays*, and *A. thaliana* (Figure 2; Additional file 1: Table S1). The rice, arabidopsis, and physcomitrella BGAL families were included because they had been studied previously and form the basis of the plant BGAL sub-family nomenclature [24,48]. The poplar and castor bean BGAL families were included as they are members of the order Malpighiales, and are relatives of flax for which whole genome sequence is available. Flax BGALs were represented in all of the BGAL sub-families, with the exception of sub-family A3, which was a bryophyte-specific cluster. In the majority of sub-families, the BGALs of flax outnumbered the BGALs of other plant species. Two exceptions to this were observed. First, flax was found to have significantly smaller representation in sub-family B (p-value < 0.01), compared to other species, with only LuBGAL43 present. By comparison, *P. trichocarpa* and *R. communis*, sequenced relatives in the same taxonomic order as flax, had five and seven BGALs, respectively, in sub-family B. Second, sub-family A2 also had a single flax representative, although, (in contrast to sub-family B) all other vascular plants in sub-family A2 were also represented by a single member. As with other vascular plants, sub-family A1 contained the largest number of LuBGAL genes, with 14 representatives, including LuBGAL1, which has been described as an important contributor to flax phloem fibre maturation [8].

### Transcript expression in public microarray datasets

**Figure 3:**
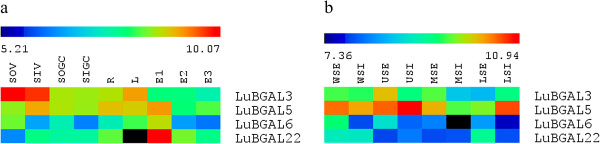
**Transcript abundance of flax BGAL genes in various tissues, from previously published microarray data sets (Nimblegen platform).** RMA-normalized, average log2 signal values of flax BGALs in various tissues were used to produce a heat map. **a**: roots (R); leaves (L); outer stem tissues at either the vegetative stage (SOV) or green capsule stage (SOGC); inner stem tissues at either vegetative stage (SIV) or green capsule stage (SIGC); and seeds 10-15 days after flowering (DAF; E1), 20-30 DAF (E2), and 40-50 DAF (E3; [33]). **b**: internal stem tissues of either the whole stem (WSI), upper stem (USI), middle stem (MSI), or lower stem (LSI); and external (i.e. phloem and cortex enriched) stem tissues of the whole stem (WSE), upper stem (USE), middle stem (MSE), and lower stem (LSE) [34].

We examined transcript expression patterns of the LuBGAL family using publicly available oligonucleotide microarray data, beginning with two experiments on a Nimblegen 25-mer oligonucleotide array (NCBI GEO experiment accessions GSE21868 [33] and GSE29345 [34]). Probes for these microarrays were designed from ESTs, and not the whole genome. Based on alignments where >90% EST length match the LuBGAL CDSs at >95% sequence identity, these microarrays contain probes for four different LuBGAL genes (*LuBGAL3*, *LuBGAL5*, *LuBGAL6*, and *LuBGAL22*). A heat map of expression values from these microarrays (Figure 3a, b) showed that *LuBGAL3* expression was enriched in the stem during vegetative growth (Figure 3a), with its highest expression in the phloem rich outer stem tissues of the upper stem (Figure 3b). *LuBGAL22* was also enriched in select tissues, and during a narrow developmental timeframe, with its greatest expression occurring in the seeds 10-15 days after flowering. Within the stem, *LuBGAL22* appeared to be more enriched in the outer stem tissues of the lower stem (Figure 3b). On the other hand, while *LuBGAL5* expression was not specific to any one tissue (Figure 3a), within the stem of vegetatively growing flax, its expression appeared enriched in the inner stem, especially in the upper stem, around the snap-point [49] where resistance to mechanical bending is first detectable, although expression was also quite high in the inner tissues of the lower stem. *LuBGAL6* did not appear to be particularly enriched in any tissue.

**Figure 4:**
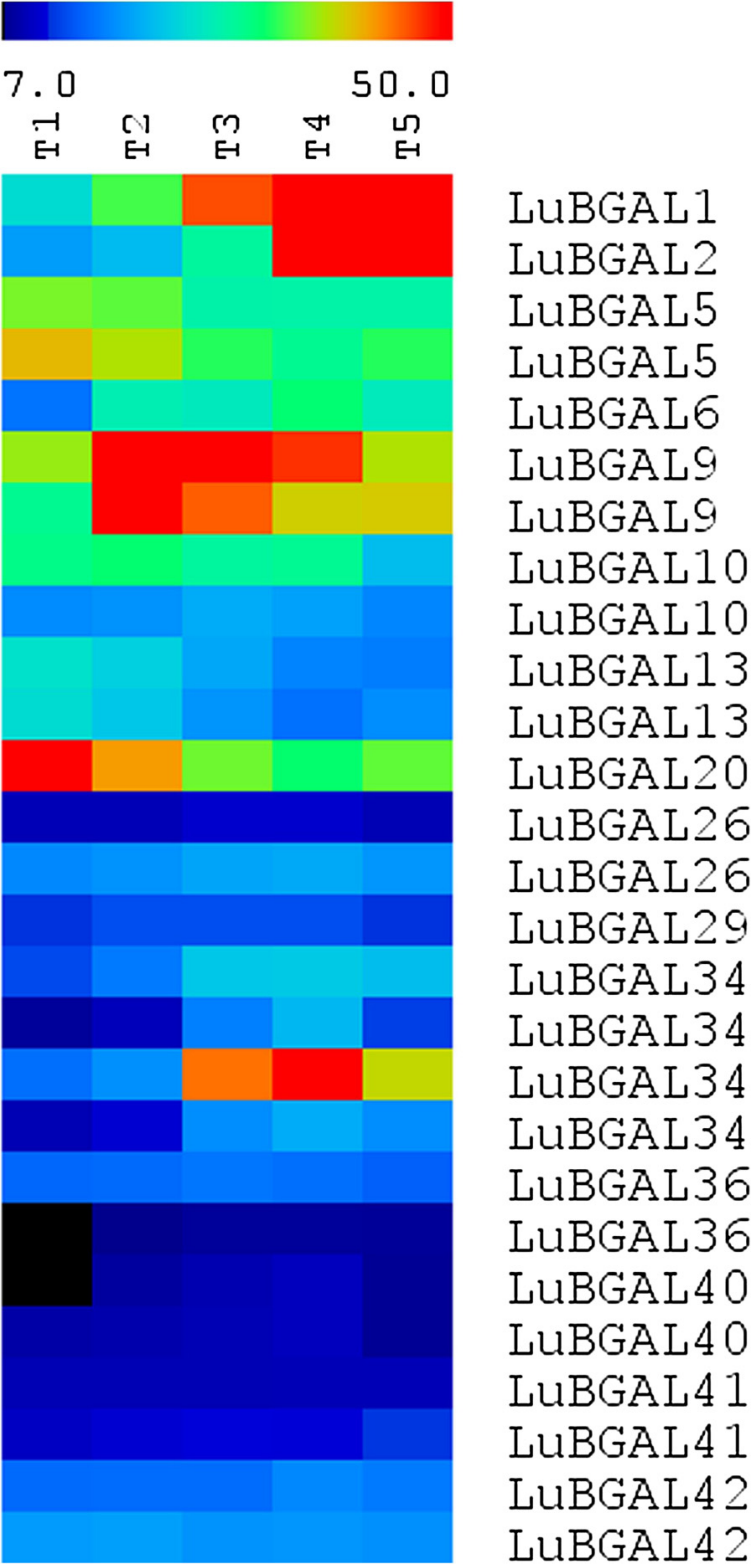
**Transcript abundance of flax BGAL genes throughout the stem, from unpublished microarray data set (Combimatrix platform).** Signal intensities were normalized as fractions of mean signal strength. The log2 signal values of the various flax BGALs were used to produce a heat map. Microarray data examined the shoot apex (T1), the snap-point through various stages of fibre development (T2, T2, and T4), and a lower portion of the stem (T5).

We further examined microarray data from a recent Combimatrix oligonucleotide array analysis of flax stem development conducted in our laboratory (manuscript in preparation). Probes for this microarray were designed from a preliminary, unpublished draft of the flax genome. After alignment to the published flax genome assembly (version 1.0) [12], 27 probes aligned to 15 distinct *LuBGAL* CDS sequences, with multiple probes corresponding to individual genes for added replication. A heat map of expression values (Figure 4) showed that a number of genes were enriched at specific developmental stages. *LuBGAL20* was clearly enriched at the shoot apex, with decreasing expression as the stem matured. *LuBGAL9* appeared enriched just above the snap-point, with expression slightly lower just below the snap-point and further down the stem, and at its lowest at the apex. *LuBGAL34* was also enriched at the snap-point, however unlike *LuBGAL9*, its expression was enriched at the lower end of this region. *LuBGAL1* and *LuBGAL2* were the last set of genes to show enrichment at a developmental stage, with their greatest expression occurring in the more mature stem tissue. While whole stem tissues were used in this assay, our previous analysis of the *LuBGAL1* promoter region provides strong evidence that the expression of this gene is specific to the phloem fibres of the stem [50].

### qRT-PCR analysis of LuBGAL expression

**Figure 5:**
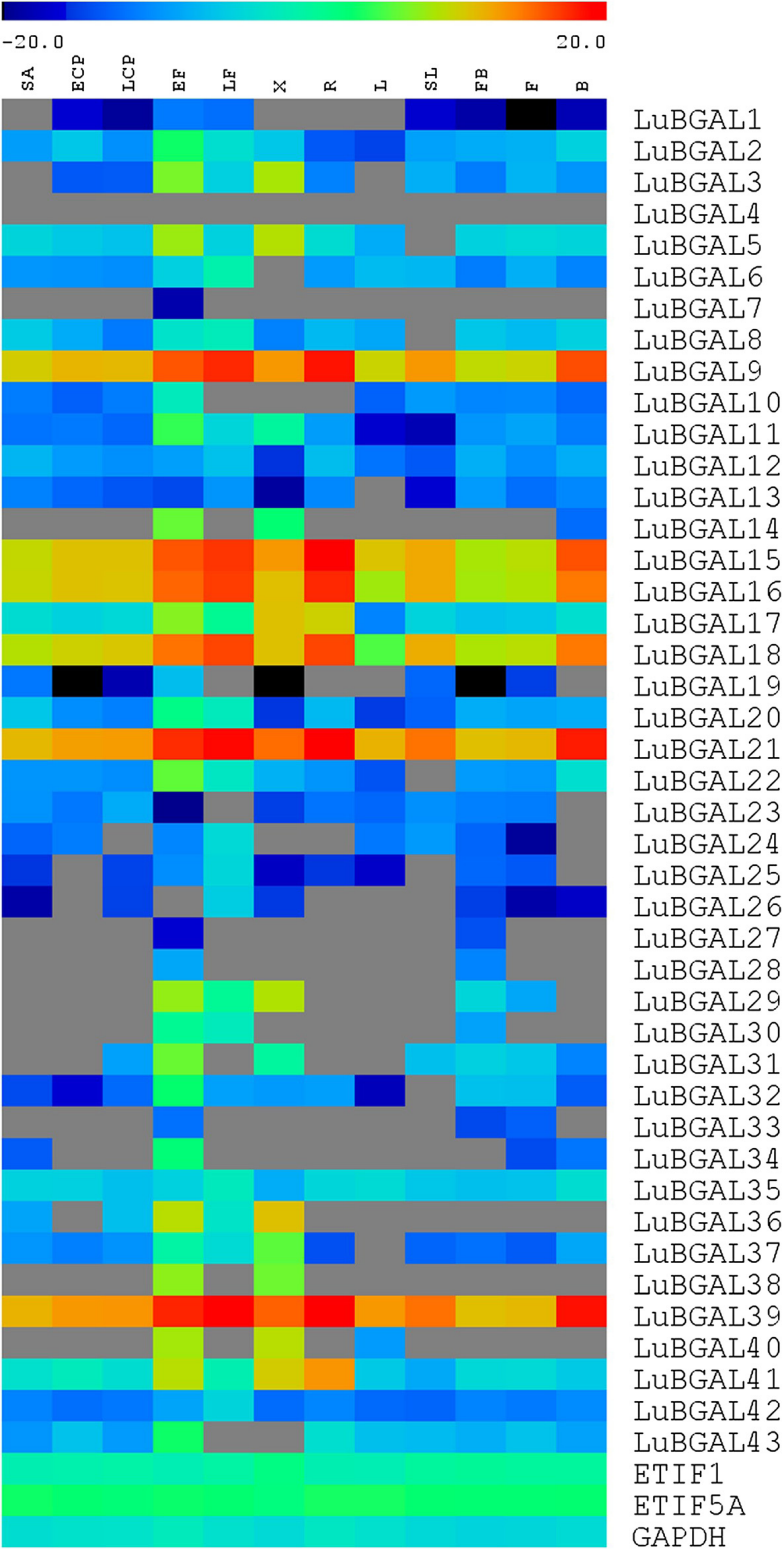
**Transcript abundance of flax BGAL genes in various tissues, by qRT-PCR (Fluidigm platform).** Expression levels (log_2_), relative to the reference genes *ETIF1* (eukaryotic translation initiation factor 1), *GAPDH* (glyceraldehyde 3-phosphate dehydrogenase), and *ETIF5A* (eukaryotic translation initiation factor 5A), were used to prepare a heat map, with blue indicating lower expression and red indicating high expression. Gray indicates no detectable expression. Tissue types analysed include: roots (R); leaves (L); senescing leaves (SL); stem apex (SA); cortical peels from vegetative stage stems (ECP) or green capsule stage stems (LCP); phloem fibres from vegetative stage stems (EF) or green capsule stage stems (LF); xylem from vegetative stage stems (X); budding flowers (FB); open flowers (F); and seed bolls from the green capsule stage (B).

Because the available microarray data sets provided transcript expression profiles for only 17 of the 43 predicted LuBGALs, we performed qRT-PCR in a Fluidigm 96*96 array, to obtain additional information about where and when members of the LuBGAL family are transcribed. With the exception of *LuBGAL20* primers, which may have amplified both *LuBGAL20* and *LuBGAL21*, primers used in the qRT-PCR analysis were verified as being gene specific following a series of BLASTN searches against the scaffolds and CDSs of the flax genome assembly. We were able to detect gene expression for 42 of the 43 LuBGAL genes in at least one of the tissues sampled (Figure 5). We could not detect expression for *LuBGAL4* in any of the tissues tested, despite identifying 34 matching ESTs in numerous databases (Table 2). This may be a false negative due to the primers; primer design options for the gene were constrained by high sequence identity to other members of the gene family and so were targeted to a putative 3’UTR of *LuBGAL4*. Maturing fibres (EF) had the greatest diversity of LuBGAL family gene expression, with 40/43 genes detected, followed by xylem, with 31/43 genes detected.

Comparing gene expression across tissues, many LuBGALs showed their highest transcript expression in tissues associated with thick secondary cell walls, i.e. the phloem fibres and xylem of vegetative stage flax stems. *LuBGAL7* expression was detected only in the early phloem fibres, whereas *LuBGALs 27*, *28*, and *38* were detected in either early phloem fibres and xylem, or in early phloem fibres and budding flowers. Among the more widely expressed genes, *LuBGALs 9*, *15*, *16*, *18*, *21*, and *39* were found to be the most highly expressed LuBGALs, with clear expression peaks in the phloem fibres of green-capsule stage flax, as well as in the roots and seed bolls. Lastly, our results confirmed that *LuBGAL1,* whose upstream genomic region was found to drive expression almost exclusively in phloem fibres [50], showed greater gene expression in the phloem fibres of vegetatively growing flax, in comparison to the other tested tissues.

## Discussion

An emerging role for β-galactosidases shows them to be important facilitators of cell wall metabolism in plants. Here, we identified 43 putative BGALs from flax, which were distributed throughout each of the previously defined BGAL sub-families of vascular plants. The relatively large number of genes in LuBGAL family, and the abundance of LuBGALs compared to BGALs of other species in each of the sub-families (Figure 2), is consistent with the recent genome duplication in the flax lineage [12]. Thus, most LuBGALs exist in pairs and likely share similar functions. Nevertheless, certain variations in the organization of the LuBGAL proteins suggest a degree of sub-functionalization and selection unique to the species, especially with regards to the reduction in the number of LuBGALs in sub-family B (Figure 2).

Aside from being the sole flax representative in sub-family B, LuBGAL43 was also the shortest predicted protein in the LuBGAL family at only 107 amino acids (Table 2), compared to the average 700-800 amino acids, and entirely lacked a GH35 active site (Additional file 3: Figure S1). While *AtBGAL1*8 and three *RcBGALs* in sub-family B likewise lacked a canonical GH35 active site, other sub-family B LuBGALs from these (and other) species had the canonical catalytic residues. Currently, no study has yet explored the biochemical function of sub-family B BGALs. Expression data have revealed that *AtBGAL7* and *AtBGAL15*, arabidopsis members of subfamily B, are expressed in flowers and pollen [23,51], whereas AtBGAL18 is expressed in seedlings and roots [51]. Similar to *AtBGAL7* and *AtBGAL15*, *OsBGALs* 5, 12, 14, and 15, the rice representatives of sub-family B, have also shown enrichment in reproductive tissues, which led to the hypothesis that the ancestor to sub-family B developed a reproductive-tissue specific role antecedent to the divergence of monocots and dicots [24]. We may further speculate that the cell wall development in flax reproductive tissues has a reduced requirement for sub-family B LuBGALs with classical GH35 active sites, as compared to vegetative tissues. Alternatively, a role for BGALs in the development of flax reproductive tissues may yet remain, but may be provided by members of different sub-families, although no individual *LuBGAL* showed enriched expression in these tissues. To better explore these possibilities, it will be important to explore the biochemical and physiological roles of sub-family B in other plant species, including testing their substrate specificity, to determine why sub-family B is not maintained in flax as in other species.

Analyses of the arabidopsis and rice BGAL families had identified 17 and 15 members respectively [23,24,48]. Our own analysis of these genomes added an additional member to each species family, both of which were under 500 amino acids in length, and both of which lacked the putative active site described by Henrissat [42]. In flax, we identified two LuBGALs, LuBGAL35 and LuBGAL43, which lacked this active site entirely, and another nine, LuBGALs 14, 20, 21, 22, 23, 24, 25, 26, and 36, which contained either partial active sites, insertions within the active sites, or a series of substitutions in key amino acids (Additional file 3: Figure S1). In Arabidopsis, BGAL activity has been characterized in AtBGAL1, AtBGAL2, AtBGAL3, AtBGAL4, AtBGAL5, AtBGAL6, AtBGAL10, and AtBGAL12 [10,11,23,48,52], all of which contain consensus GH35 active sites. The radish RsBGAL1, characterized as a BGAL hydrolyzing β-(1 → 3)- and β-(1 → 6)-galactosyl residues, also contains the consensus GH35 active site [53], as does a recently characterized chickpea BGAL [54], and a number of other cloned BGALs [55,56]. In fact, all biochemically verified plant BGALs reported to date contain the consensus GH35 active site. Therefore, the absent, partial, and altered GH35 active sites in predicted LuBGAL proteins may indicate a shift in substrate specificity and/or enzyme kinetics, if not a complete lack of enzymatic activity.

LuBGALs 20-25 make up the entirety of sub-family A5 in flax, which, in additional to being composed entirely of LuBGALs with non-conserved GH35 active sites, is also of interest due to the manner in which the sub-family has expanded in comparison to related species (p-value < 0.01). Rice, arabidopsis, poplar, and castor each contain a single member in sub-family A5, whereas flax contained six members. *Arabidopsis lyrata*, *Medicago truncatula*, *Vitis vinifera*, *Aquilegia coerulea*, *Cucumis sativus*, *Prunus persica*, *Mimulus guttatus*, *Brachypodium dystachion*, *Setaria italica*, *Sorghum bicolor*, *Zea mays*, *Nasturtium microphyllum*, *Solanum lycopersicum*, and *Pyrus communis* have also been described as containing a single sub-family A5 representative [10]. Exceptions occur in *Citrus sinensis*, *Citrus clementina*, *Glycine max*, and *Eucalyptus grandis*, where two members of sub-family A5 were recorded [11]. With regards to the changes in its putative GH35 active site, the shared mutations observed in LuBGAL22 and LuBGAL24, as well as in LuBGAL20 and LuBGAL21, would suggest that the divergence in sequence from sub-family A5 orthologs predates the last genome duplication. In addition to the changes in the GH35 active site, LuBGAL22, LuBGAL24, and LuBGAL25 are also of uncommon size. LuBGAL22 and LuBGAL24 are over 1300aa in length, and, in the case of LuBGAL24, containing additional N-terminal copper oxidase domains, possibly the result of a gene fusion. In contrast, LuBGAL25 appears truncated, coding for a protein 297aa in length. AtBGAL10, the sole arabidopsis member of sub-family A5, has been described as the main xyloglucan β-galactosidase of arabidopsis, where T-DNA insertions in *AtBGAL10* have led to a 90% decrease in BGAL activity against XLLG substrates, where G refers to an unsubstituted glucose residue of the xyloglucan backbone, X refers to a glucose substituted with α-D-Xylp sidechain, and L refers to a glucose residue substituted with β-D-Galp-(1 → 2)- α-D-Xylp sidechain [10]. Expression of *AtBGAL10* was observed to be quite strong in developing flowers, the columella cells and elongation zone of the roots, as well as the in the developing vasculature, trichomes, and guard cells of the leaves, all of which are areas of intense cell wall remodelling for cell division and expansion [10]. *LuBGAL21,* too, was strongly expressed in roots, and developing seed bolls. *LuBGAL22* was observed to be expressed strongly in seeds early in development (Figure 3a), while *LuBGAL20* appeared to be strongly expressed in the shoot apex (Figure 4), all of which might indicate a role in cell division. The remainder of the sub-family A5 *LuBGAL*s were primarily expressed in vegetatively growing phloem fibres (Figure 5), which exhibit secondary cell wall deposition as opposed to cell division or elongation.

BGAL sub-family A1 is the best studied of all the BGALs, having been described as encoding exogalactanases, generally hydrolyzing β-(1,3)- and β-(1,4)-linked galacto-oligosaccharides of the cell wall [23,52], and, in the case of AtBGAL12, additionally hydrolyzing β-(1,6)-galacto-oligosaccharides [48]. In flax, LuBGAL1 has previously been posited to play an important role in the degradation of high molecular weight poly-galactans in the secondary cell walls of phloem fibres. When silenced, the reduction in LuBGAL1 activity (and possible reduction in LuBGAL2 activity) leads to retention of these pectic galactans, which apparently results in reduced crystallization of cellulose, thus reducing the structural integrity of flax stems [8]. Further characterization of the LuBGAL1 promoter region supports high specificity of expression in phloem fibres [53], which our expression analyses reported here have again confirmed (Figures 4 and 5). It appears likely that other LuBGALs in sub-family A1 share similar functions as LuBGAL1, based on conservation of their coding sequences and similarity of their expression patterns. Sequences sharing the greatest similarity to LuBGAL1 exhibited a very similar pattern of expression: *LuBGALs 2, 3, 7, 6, and 5*, which comprised the same branch of sub-family A1 as LuBGAL1, consistently showed greater expression in tissues rich in secondary cell walls, be it phloem fibres or xylem (Figure 5). The sole exception was *LuBGAL4*, for which no expression has been detected in either microarray or qRT-PCR. In some cases, such as *LuBGAL5*, expression was also strong in developing seeds (Figure 3a), however this overlap with reproductive tissues has been likewise observed in *LuBGAL1*[50]. Perhaps unsurprisingly, *LuBGAL2*, the most similar paralog of *LuBGAL1*, appears to follow the same expression pattern as it relates to developmental stages in the stem (Figure 4), being expressed just below the snap-point, where the secondary cell walls of phloem fibres begin to exhibit the shift from a galactan rich Gn-layer to a more cellulose rich G-layer [49]. The other major group within sub-family A1 (*LuBGALs 8*, *9*, *12*, *13*, *14*, *15*, and *16*) appear more varied in expression. While some members, such as *LuBGALs 8*, *11*, and *14* are particularly enriched in fibres and xylem, others, such as *LuBGALs 9*, *15*, and *16*, are more strongly expressed throughout the plant, with greater expression in roots (Figure 5). We note that these genes do also show expression in stem tissues, however, expression appears restricted to different developmental stages (Figure 4). In the case of *LuBGAL9*, expression was observed to occur above the snap-point, which, in the case of phloem fibres, is where cells are still undergoing cell elongation [49]. All told, the general expression pattern of this branch of sub-family A1 suggests that their function has diverged further from *LuBGAL1* than its immediate sisters.

BGAL sub-family C2 is also a well-characterized group of BGALs. Mutations in *AtBGAL6* (*MUM2*) inhibit the secretion of pectinaceous seed mucilage during hydration [11]. The LuBGALs with the most sequence similarity to *AtBGAL6* were *LuBGALs 34-37*, and their expression was detected in seed capsules, with the exception of *LuBGAL36.* Greater characterization will be required to determine whether these genes play a similar role in seed coat development.

The remainder of the flax BGALs were observed to express themselves in a variety of tissues, with over half observed to be most strongly expressed in the phloem fibres of vegetatively growing flax stems, relative to the other examined tissues (Figure 5). The maturation of flax phloem fibres involves the deposition and later degradation of a large galactan-rich polysaccharide [57], which is likely one of the main substrates of these BGAL proteins.

It should be noted that slight differences in expression patterns were observed when comparing genes across Nimblegen, Combimatrix, and Fluidigm platforms. We attribute this to differences in binding efficiencies between cDNA and probes of the microarrays, and cDNA, primers, and hydrolysis probes of in the qPCR analyses. Additionally, each platform utilized a different cultivar of flax, grown under dissimilar environmental conditions. Therefore, we attempted to focus not on minor differences in expression between tissues, but rather on the larger differences.

## Conclusion

Forty-three putative BGAL genes were identified in the genome of *Linum usitatissimum*. Clustered into eight distinct sub-families, the flax BGAL family was observed to be large in comparison to other sequenced species, with distinct differences in family composition not observed in related species of the order Malpighiales, including a reduction in gene representation in sub-family B, an increased representation in sub-family A5, and many alterations to the typically consensus GH35 active site in a large number of LuBGALs. Using a combination of EST, microarray, and qRT-PCR data, we were able to detect the expression of each member of the LuBGAL family. Almost every *LuBGAL* was expressed in the fibres, the majority of which were predominantly expressed in fibres, compared to other tissues. This suggests that the expansion of the LuBGAL family played an important role in the development of this species as a fibre crop. Further characterization will be necessary to better elucidate their precise function in flax development.

## Competing interests

The authors declare that they have no competing interests.

## Authors’ contributions

NH performed the database searches, bioinformatics analyses, qRT-PCR, data analysis, and drafted the manuscript. MKD designed, coordinated, and supervised the study. Both authors have participated in writing and revising the manuscript, and have read and approved the final version of the manuscript.

## Supplementary Material

Additional file 1: Table S1 Genomic loci and accessions of analysed BGALs. Genome assemblies for plant species can be obtained from Phytozome (version 8.0) [15]. Click here for file

Additional file 2: Table S2 Primers and hydrolysis probes used in qRT-PCR analysis. Oligonucleotide primer sequences and probes for LuBGAL genes were obtained from the Universal Probe Library Assay Design Center [38]. Click here for file

Additional file 3: Figure S1 Putative GH35 active site in various plant species. The GH35 active site [42], was identified by searching for the consensus sequence G-G-P-[LIVM](2)-x(2)-Q-x-E-N-E-[FY]. Gaps or missing sequence are denoted by dashes ‘-‘. Residues conserved amidst 90% of the sequences are highlighted. The flax sequences are named LuBGAL, and numbered according to Tables 1 and 2. *Arabidopsis thaliana* sequences are indicated as AtBGAL, and numbered according to existing designations [24]. *Oryza sativa* sequences are indicated as OsBGAL, and numbered according to existing designations [25]. Genomic loci corresponding to these sequences are presented in Table 1 and Additional file 1: Table S1. Click here for file
