# Supplementary material for: Genomic and expression analysis of the flax (Linum usitatissimum) family of glycosyl hydrolase 35 genes
Source: BMC Genomics. 2013 May 23;14:344. doi: 10.1186/1471-2164-14-344 (PMC3673811; doi:10.1186/1471-2164-14-344)
Supplement: Additional file 1: Table S1 — Genomic loci and accessions of analysed BGALs. Genome assemblies for plant species can be obtained from Phytozome (version 8.0) [15]. [file 1471-2164-14-344-S1.doc]

| **Species** | **Gene** | **Locus** | **GenbankAccession** |
| --- | --- | --- | --- |
| *Homo sapiens* | GLB1 |  | NP_000395 |
| *Arabidopsis thaliana* | AtBGAL01 | At3G13750 |  |
|  | AtBGAL02 | At3G52840 |  |
|  | AtBGAL03 | At4G36360 |  |
|  | AtBGAL04 | At5G56870 |  |
|  | AtBGAL05 | At1G45130 |  |
|  | AtBGAL06 | At5G63800 |  |
|  | AtBGAL07 | At5G20710 |  |
|  | AtBGAL08 | At2G28470 |  |
|  | AtBGAL09 | At2G32810 |  |
|  | AtBGAL10 | At5G63810 |  |
|  | AtBGAL11 | At4G35010 |  |
|  | AtBGAL12 | At4G26140 |  |
|  | AtBGAL13 | At2G16730 |  |
|  | AtBGAL14 | At4G38590 |  |
|  | AtBGAL15 | At1G31740 |  |
|  | AtBGAL16 | At1G77410 |  |
|  | AtBGAL17 | At1G72990 |  |
|  | AtBGAL18 | At2G04060 |  |
| *Physcomitrella patens* | PpBGAL01 | Pp1s24_113V6.1 |  |
|  | PpBGAL02 | Pp1s189_71V6.1 |  |
|  | PpBGAL03 | Pp1s10_144V6.1 |  |
|  | PpBGAL04 | Pp1s49_266V6.1 |  |
|  | PpBGAL05 | Pp1s52_244V6.1 |  |
|  | PpBGAL06 | Pp1s189_65V6.1 |  |
| *Populus trichocarpa* | PtBGAL01 | Potri.017G057900 |  |
|  | PtBGAL02 | Potri.009G012400 |  |
|  | PtBGAL03 | Potri.011G044300 |  |
|  | PtBGAL04 | Potri.009G134400 |  |
|  | PtBGAL05 | Potri.004G174800 |  |
|  | PtBGAL06 | Potri.013G105100 |  |
|  | PtBGAL07 | Potri.002G080700 |  |
|  | PtBGAL08 | Potri.005G180600 |  |
|  | PtBGAL09 | Potri.005G069200 |  |
|  | PtBGAL10 | Potri.003G040000 |  |
|  | PtBGAL11 | Potri.003G037000 |  |
|  | PtBGAL12 | Potri.T154000 |  |
|  | PtBGAL13 | Potri.001G025700 |  |
|  | PtBGAL14 | Potri.001G025800 |  |
|  | PtBGAL15 | Potri.006G139100 |  |
|  | PtBGAL16 | Potri.018G062800 |  |
|  | PtBGAL17 | Potri.004G159800 |  |
|  | PtBGAL18 | Potri.007G018100 |  |
|  | PtBGAL19 | Potri.005G232600 |  |
|  | PtBGAL20 | Potri.003G038500 |  |
|  | PtBGAL21 | Potri.001G200400 |  |
|  | PtBGAL22 | Potri.006G144500 |  |
|  | PtBGAL23 | Potri.007G099800 |  |
| *Oryza sativa* | OsBGAL01 | LOC_Os03g06940 |  |
|  | OsBGAL02 | LOC_Os06g37560 |  |
|  | OsBGAL03 | LOC_Os01g39830 |  |
|  | OsBGAL04 | LOC_Os01g65460 |  |
|  | OsBGAL05 | LOC_Os01g34920 |  |
|  | OsBGAL06 | LOC_Os05g35360 |  |
|  | OsBGAL07 | LOC_Os02g12730 |  |
|  | OsBGAL08 | LOC_Os03g15020 |  |
|  | OsBGAL09 | LOC_Os05g46200 |  |
|  | OsBGAL10 | LOC_Os08g43570 |  |
|  | OsBGAL11 | LOC_Os09g36810 |  |
|  | OsBGAL12 | LOC_Os10g18400 |  |
|  | OsBGAL13 | LOC_Os12g24170 |  |
|  | OsBGAL14 | LOC_Os10g19960 |  |
|  | OsBGAL15 | LOC_Os06g42310 |  |
|  | OsBGAL16 | LOC_Os05g35370 |  |
| *Ricinus communis* | RcBGAL01 | 28076.m000428 |  |
|  | RcBGAL02 | 29917.m001961 |  |
|  | RcBGAL03 | 28694.m000663 |  |
|  | RcBGAL04 | 50666.m000014 |  |
|  | RcBGAL05 | 29739.m003718 |  |
|  | RcBGAL06 | 29739.m003719 |  |
|  | RcBGAL07 | 29739.m003720 |  |
|  | RcBGAL08 | 29739.m003740 |  |
|  | RcBGAL09 | 29739.m003723 |  |
|  | RcBGAL10 | 29815.m000493 |  |
|  | RcBGAL11 | 29904.m002899 |  |
|  | RcBGAL12 | 59437.m00005 |  |
|  | RcBGAL13 | 28694.m000675 |  |
|  | RcBGAL14 | 29912.m005323 |  |
|  | RcBGAL15 | 29912.m005324 |  |
|  | RcBGAL16 | 30131.m007094 |  |
|  | RcBGAL17 | 30170.m014108 |  |
|  | RcBGAL18 | 29648.m002008 |  |
|  | RcBGAL19 | 30193.m000721 |  |
|  | RcBGAL20 | 30074.m001370 |  |
|  | RcBGAL21 | 30193.m000718 |  |
| *Zea mays* | ZmBGAL01 | AC199908.4 |  |
|  | ZmBGAL02 | AC234152.1 |  |
|  | ZmBGAL03 | GRMZM2G027385 |  |
|  | ZmBGAL04 | GRMZM2G038281 |  |
|  | ZmBGAL05 | GRMZM2G071883 |  |
|  | ZmBGAL06 | GRMZM2G073584 |  |
|  | ZmBGAL07 | GRMZM2G081583 |  |
|  | ZmBGAL08 | GRMZM2G121495 |  |
|  | ZmBGAL09 | GRMZM2G127123 |  |
|  | ZmBGAL10 | GRMZM2G130375 |  |
|  | ZmBGAL11 | GRMZM2G151122 |  |
|  | ZmBGAL12 | GRMZM2G153200 |  |
|  | ZmBGAL13 | GRMZM2G162238 |  |
|  | ZmBGAL14 | GRMZM2G164676 |  |
|  | ZmBGAL15 | GRMZM2G175779 |  |
|  | ZmBGAL16 | GRMZM2G178106 |  |
|  | ZmBGAL17 | GRMZM2G386824 |  |
|  | ZmBGAL18 | GRMZM2G417455 |  |
|  | ZmBGAL19 | GRMZM2G465617 |  |
|  | ZmBGAL20 | GRMZM5G828603 |  |

**Table S1 Genomic loci and accessions of analysed BGALs.** Genome assemblies for plant species can be obtained from Phytozome (version 8.0) [14].
