# Supplementary material for: Genomic and expression analysis of the flax (Linum usitatissimum) family of glycosyl hydrolase 35 genes
Source: BMC Genomics. 2013 May 23;14:344. doi: 10.1186/1471-2164-14-344 (PMC3673811; doi:10.1186/1471-2164-14-344)
Supplement: Additional file 2: Table S2 — Primers and hydrolysis probes used in qRT-PCR analysis. Oligonucleotide primer sequences and probes for LuBGAL genes were obtained from the Universal Probe Library Assay Design Center [38]. [file 1471-2164-14-344-S2.doc]

| **Gene** | **Forward Primer** | **Reverse Primer** | **Hydrolysis Probe** |
| --- | --- | --- | --- |
| LuBGAL01 | agacccatttctcaatgtcca | cgatgagttttccattgacg | 18 |
| LuBGAL02 | ctttggaaatggccagtatga | cgttttgcaatcaggcagta | 33 |
| LuBGAL03 | cgtaaagccataatcgtcaatg | ggagtgcttctggggtagtg | 2 |
| LuBGAL04 | ccacagtcatgggaatagcc | cttcatgtacaatacccattccac | 36 |
| LuBGAL05 | atgatggcaggtgggactt | cacccttgagaccaatcttgt | 29 |
| LuBGAL06 | catcccgaagcttcacaataa | tggaggaaatcagaatggaga | 5 |
| LuBGAL07 | taccacgtccctcgttcg | cggattcctcaaaaacgact | 139 |
| LuBGAL08 | gaggagaggcgttgaatttg | tcttcagcccactcaacaga | 19 |
| LuBGAL09 | gctgtggtctcctctgatcc | tgagaacacatgagcctgttg | 48 |
| LuBGAL10 | cgttgtccagaatgcttgc | gcagccatcgaaagagga | 142 |
| LuBGAL11 | ttggcctgcctatctctcc | cttcctctgtagtcacagttctcg | 46 |
| LuBGAL12 | ggtaaatggtccctgcaagtt | aggcgcctttcacgtactc | 110 |
| LuBGAL13 | taagtggttccccgcaaat | aggcgcctttcaagtactca | 110 |
| LuBGAL14 | gcctaccaacaggattgtgc | tttcgtagcagattttgtgtcg | 137 |
| LuBGAL15 | gcaagctgtttggtgtacctg | tccagttttgacagccatgt | 26 |
| LuBGAL16 | tgaactttacaagtagggccagt | atgaccagtgcccacttacc | 29 |
| LuBGAL17 | actgaaaattggagcggatg | atgcaaggtcttccacaggt | 119 |
| LuBGAL18 | ttatatgtaccatggtgggactaact | gtggcgataaagggtccac | 12 |
| LuBGAL19 | ttgcaagtagacgaccacca | gccaagtaaagcaatgaggtg | 153 |
| LuBGAL20 | gatggccgatctctcatcat | agtgaatggaagcggaaatg | 6 |
| LuBGAL21 | cgctagactgggatgcttctt | tcccaccctctccatagaact | 39 |
| LuBGAL22 | cgatcattgttcaagccatc | gtcgccgcctatctcctc | 88 |
| LuBGAL23 | accttcagaggtgcccaaa | ggctcatccacaatcacctt | 110 |
| LuBGAL24 | gaagtcgtggacggcagtat | gtccgcacgttccacatt | 21 |
| LuBGAL25 | tcggtggaaaatgaataccaat | catattagcagcccagtaagca | 60 |
| LuBGAL26 | ctcttcatggcttgctcgat | gaattgagccggagaacaga | 34 |
| LuBGAL27 | gcttggtacacaaccaagatacaa | tgccctaaacttgccacttc | 76 |
| LuBGAL28 | aactgttgagttcaggggaaag | tggcagaatgctaacagagg | 8 |
| LuBGAL29 | ccagccggaatacagtgg | cggcgacaatacgaagaag | 153 |
| LuBGAL30 | cgttgccgtcttcctcatac | ttattgtcatcgtgagcattgac | 131 |
| LuBGAL31 | gctaacttcaagggccagaa | aatcaggaaggacgctgatg | 69 |
| LuBGAL32 | cccctgtctccaagcaagt | atgggacatcgcacgaat | 150 |
| LuBGAL33 | tttgttgagcgtctctgtagga | taaagccgctcgctcttg | 50 |
| LuBGAL34 | cggcaaatatatagggaactcg | tgacacagtgagattggagctt | 6 |
| LuBGAL35 | gaagggaaactgtgcagcat | gcatttcgaaacacaactgc | 129 |
| LuBGAL36 | ttttcttgctttgctgttgc | cttcatcttcgccttcttgtc | 143 |
| LuBGAL37 | aacgtcgaggcagcattc | acataacccacgggacttca | 44 |
| LuBGAL38 | agacgtttaacggagccaac | cttgtcccgttttctttcca | 137 |
| LuBGAL39 | agctggagttcggagttcac | aggaatggaaaggaaatgagg | 143 |
| LuBGAL40 | ccctttcattgaagctgagtg | atgtctgggacatcatgcaa | 157 |
| LuBGAL41 | aacatcccccttgtaactaaatagaat | gcttcaggacacacacctatga | 153 |
| LuBGAL42 | tggagttgaatccgaaaacc | gtcacgtatagcatgaaaccaaa | 123 |
| LuBGAL43 | cggtcgagcaatcaccat | gcttcgtgggtaatggactg | 101 |

**Table S2 Primers and hydrolysis probes used in qRT-PCR analysis.** Oligonucleotide primer sequences and probes for LuBGAL genes were obtained from the Universal Probe Library Assay Design Center [37].
