# Supplementary material for: Genomic and expression analysis of the flax (Linum usitatissimum) family of glycosyl hydrolase 35 genes
Source: BMC Genomics. 2013 May 23;14:344. doi: 10.1186/1471-2164-14-344 (PMC3673811; doi:10.1186/1471-2164-14-344)
Supplement: Additional file 3: Figure S1 — Putative GH35 active site in various plant species. The GH35 active site [42], was identified by searching for the consensus sequence G-G-P-[LIVM](2)-x(2)-Q-x-E-N-E-[FY]. Gaps or missing sequence are denoted by dashes ‘-‘. Residues conserved amidst 90% of the sequences are highlighted. The flax sequences are named LuBGAL, and numbered according to Tables 1 and 2. Arabidopsis thaliana sequences are indicated as AtBGAL, and numbered according to existing designations [24]. Oryza sativa sequences are indicated as OsBGAL, and numbered according to existing designations [25]. Genomic loci corresponding to these sequences are presented in Table 1 and Additional file 1: Table S1. [file 1471-2164-14-344-S3.pdf]

```
                   10        20        30        40
          ....|....|....|....|....|....|....|....|
AtBGAL01  RLFESQGG-PIILSQ--------------IENEYGPMEYE
AtBGAL02  KLFETQGG-PIILSQ--------------IENEYGPMQWE
AtBGAL03  NLFESQGG-PIILSQ--------------IENEYGRQGQL
AtBGAL04  KLFQTQGG-PIILAQ--------------IENEYGPVEWE
AtBGAL05  RFFASQGG-PIILSQ--------------IENEFEPDLKG
AtBGAL06  GLYASQGG-PIILSQ--------------IENEYANVEGA
AtBGAL07  KLFASQGG-PIILAQ--------------IENEYGNVISS
AtBGAL08  KLYASQGG-PIILSQ--------------IENEYGNIDSA
AtBGAL09  KLFCWQGG-PIIMLQ--------------IENEYGDVEKS
AtBGAL10  KLFAPQGG-PIILSQ--------------VENEYGYYEKD
AtBGAL11  RLFASQGG-PIILGQ--------------IENEYSAVQRA
AtBGAL12  KLFETQGG-PIILSQ--------------IENEYGPIEWE
AtBGAL13  KLFASQGG-PIILGQ--------------IENEYSAVQRA
AtBGAL14  KLFASQGG-PIILGQ--------------IENEYNAVQLA
AtBGAL15  KLFASQGG-PIILAQ--------------IENEYGNVIGS
AtBGAL16  NLYASQGG-PIILSQ--------------IENEYGMVGRA
AtBGAL17  PLLYSNGG-PVIMVQ--------------IENEYGSYGND
AtBGAL18  ----------------------------------------
LuBGAL01  QLFEPQGG-PIILSQ--------------IENEYGPIEWE
LuBGAL02  ELFEPQGG-PIILSQ--------------IENEYGPIEWE
LuBGAL03  KLFEPQGG-PIILSQ--------------IENEYGPVEYE
LuBGAL04  KLFEPQGG-PIILSQ--------------IENEYGPVEYE
LuBGAL05  RLFETQGG-PIILSQ--------------IENEYGPMEYE
LuBGAL06  RLFENQGG-PIILSQ--------------IENEYGPMENE
LuBGAL07  RLFENQGG-PIILSQ--------------IENEYGPMEYE
LuBGAL08  KLFASQGG-PIILSQ--------------IENEYGPESKA
LuBGAL09  KLFASQGG-PIILSQ--------------IENEYGPESKA
LuBGAL10  NLFASQGG-PIILAQ--------------VENEYGNVEWA
LuBGAL11  QLFASQGG-PIILAQ--------------VENEYGNVQAS
LuBGAL12  SLFESQGG-PIILSQ--------------IENEYGAQSKM
LuBGAL13  SLFESQGG-PIILSQ--------------IENEYGAQSKM
LuBGAL14  SLFESQGG-PIILSK--------------IENEYGAQSKM
LuBGAL15  NLFESQGG-PIILSQ--------------IENEYGVQSKL
LuBGAL16  NLFESQGG-PIILSQ--------------IENEYGVQSKL
LuBGAL17  KLYASQGG-PIILSQ--------------IENEYGNVDAA
LuBGAL18  KLYASQGG-PIILSQ--------------IENEYGNVDAA
LuBGAL19  QLFSWQGG-PIIMLQ--------------IENEYGNIESS
LuBGAL20  KLFASQG------DWAA-------ASSLQVENEYGYYEQF
LuBGAL21  KLFASQGASSSSLDWD--------ASSLQVENEYGYYEQF
LuBGAL22  KLFASQGG-PIILAQASKT-----ITQETVENEYGFYEQY
LuBGAL23  AAEWNYGGIPVWLHYIPGTVFRTDNTNFKVENEYQYYEQF
LuBGAL24  KLFASQGG-PIILAQASKT-----IIQEKVENEYWLYEQY
LuBGAL25  ASQGDNG--PIILAQAS-----TKQS-FSVENEYQYYEQF
LuBGAL26  NLFASQGG-PIIMSQ--------------INTCNGRHCAN
LuBGAL27  KLFASQGG-PIIMSQ--------------IENEYANVERA
LuBGAL28  RLFASQGG-PIIMSQ--------------VENEYGNVEGV
LuBGAL29  KLFAGQGG-PIILAQ--------------IENEYNTVQLA
LuBGAL30  KLFAGQGG-PIILAQ--------------IENEYNTVQLA
LuBGAL31  NLFAEQGG-PIILAQ--------------IENEYNTVQLA
LuBGAL32  NLFAEQGG-PIILAQ--------------IENEYNTVQLA
LuBGAL33  NLFASQGG-PIILSQ--------------IENEYTNVESA
LuBGAL34  GLFASQGG-PIILAQ--------------IENEYQNIEAA
LuBGAL35  ----------------------------------------
LuBGAL36  GLFASDGG-PILLAQ--------------IENVYQNVEAA
LuBGAL37  GLFASQGG-PIILAQ--------------IENEYQNVEAA
LuBGAL38  NLYASQGG-PIILSQ--------------VENEYQTIESA
LuBGAL39  NLYASQGG-PIILSQ--------------VENEYQTIESA
LuBGAL40  NLYASQGG-PIILTQ--------------IENEYGTYEGA
LuBGAL41  TLLYNNGG-PVIMVQ--------------IENEFGSYGDD
LuBGAL42  PLLYNNGG-PIIMVQ--------------IENEFGSYGDD
LuBGAL43  ----------------------------------------
OsBGAL01  NLFASQGG-PIILSQ--------------IENEYGPEGKE
OsBGAL02  GLFEWQGG-PIILAQ--------------VENEYGPMESV
OsBGAL03  GLFEWQGG-PIILSQ--------------IENEFGPLEWD
OsBGAL04  QFFASQGG-HIILAQ--------------VENEYGDMEQA
OsBGAL05  KMFAEQGG-PIILAQ--------------IENEYGNIMGK
OsBGAL06  GLYYPQGG-PIIISQ--------------IENEYQMIEPA
OsBGAL07  GLFEWQGG-PIIMSQ--------------VENEFGPMESV
OsBGAL08  GLYASQGG-PIILSQ--------------IENEYGNIDSA
OsBGAL09  PLLYSNGG-PIIMVQ--------------IENEFGSFGDD
OsBGAL10  KLFASQGG-PIILAQ--------------IENEYQHLEVA
OsBGAL11  EMFAPQGG-PIILSQ--------------IENEYGNIKKD
OsBGAL12  NMFAGQGG-PIILAQ--------------IENEYGNVMGQ
OsBGAL13  KLYSWQGG-PIILQQ--------------IENEYGNIQGN
OsBGAL14  NMFAGQGG-PIILAQ--------------IENEYGYTMLQ
OsBGAL15  NMFAGQGG-PIILAQ--------------IENEYGNIMGQ
OsBGAL16  ----------------------------------------
```
